# Risk of Low Energy Availability among Female and Male Elite Runners Competing at the 26th European Cross-Country Championships

**DOI:** 10.3390/nu13030873

**Published:** 2021-03-07

**Authors:** Filipe Jesus, Inês Castela, Analiza M Silva, Pedro A. Branco, Mónica Sousa

**Affiliations:** 1 Nutrition & Metabolism, NOVA Medical School, Faculdade de Ciências Médicas, Universidade NOVA de Lisboa, Campo dos Mártires da Pátria, 1169-056 Lisboa, Portugal; filipejesus@fmh.ulisboa.pt (F.J.); ines.castela@nms.unl.pt (I.C.); 2 Exercise and Health Laboratory, CIPER, Faculdade de Motricidade Humana, Universidade de Lisboa, Estrada da Costa, 1499-002 Cruz-Quebrada, Portugal; analiza@fmh.ulisboa.pt; 3 CINTESIS, NOVA Medical School, NMS, Universidade Nova de Lisboa, 1169-056 Lisboa, Portugal; 4 Medical & Anti-Doping Commission, European Athletics, CH-1003 Lausanne, Switzerland; pedro.branco@european-athletics.org

**Keywords:** nutrition, exercise, questionnaire, injuries, gastrointestinal function, menstrual function

## Abstract

Low energy availability (LEA) causes impaired physiological functioning. Cross-country running is a weight-sensitive sport, making athletes more prone to LEA. We aimed to estimate the prevalence of elite European cross-country athletes at risk of LEA using the LEA in Females Questionnaire (LEAF-Q) and to analyze demographic and physical characteristics that are associated with LEA. Athletes ≥ 18 years competing at the 26th European Cross-Country Championships (*n* = 602) were invited to complete a questionnaire (sociodemographic, training, anthropometric characteristics, and LEAF-Q). A total of 207 valid surveys were collected (83 females, 22.1 (4.0) years, and 124 males, 22.3 (4.1) years), and 16 surveys were excluded. A high prevalence of athletes at risk of LEA (64.3%) was observed, being higher in females than in males (79.5 and 54.0% respectively, *p* < 0.001). More than half of athletes (54.1%, *n* = 112) reported bowel movements once a week or more rarely, while 33 female athletes (41.3%) did not report normal menstruation. Overall, cross-country athletes are at high risk of LEA. Moreover, a high prevalence of gastrointestinal and menstrual impairments was reported. Hence, athletes should be followed by multidisciplinary teams to inform, prevent, and treat LEA and its effects.

## 1. Introduction

Energy availability (EA) is defined as the difference between energy intake (EI) and exercise energy expenditure (EEE) in relation to fat-free mass (FFM) [1]. Values of EA below 30 kcal/kg FFM/day have been identified as a threshold where the luteinizing hormone is impaired [2]. Hence, it has been established that values below this threshold refer to clinical low EA (LEA), whereas subclinical LEA ranges from clinical LEA to optimal levels of EA (i.e., 45 kcal/kg FFM/day for female and 40 kcal/kg FFM/day for male athletes) [2]. Whereas the health and performance outcomes described by De Souza et al. [3] for the Female Athlete Triad model (i.e., bone health, menstrual function, and disordered eating in female athletes) and by Mountjoy et al. [4] for Relative Energy Deficiency in Sports (RED-S) (i.e., 10 health outcomes and 10 exercises consequences in males and females, including gastrointestinal function impairment and injuries) may differ, LEA is the underlying cause for both [5]. Moreover, risk factors such as low weight and fat mass have been associated with LEA or its surrogates (e.g., amenorrhea) [2]. Hence, the identification of the risk factors, and thus, athletes at risk of LEA, is the first step to prevent the development or to initiate the treatment of the consequences of LEA [4].

Although the assessment of EA is fundamental, no reference methodology is yet available. In fact, even the more commonly used methods to assess EA (such as food records to assess EI and accelerometers or exercise records to assess EEE) may present problems regarding reliability, validity, and burden upon the athletes [6]. On the other hand, questionnaires have been developed as more practical tools to estimate the risk of LEA. One example is the Low Energy Availability in Females Questionnaire (LEAF-Q), which was developed with the specific goal of identifying female athletes at risk of LEA [7]. Although the version of the questionnaire for male athletes is still under development [4], the LEAF-Q has already been used in male athletes, excluding the menstrual function section and using a specific risk score [8].

Athletics’ events, such as cross-country, are characterized by high training loads which, in turn, lead to higher physiological demands [2,9]. Moreover, cross-country is identified as a weight-sensitive sport. These sports are characterized by the strong association between body weight and performance, with an increased body weight signifying an increased endeavor by gravity [10]. Hence, cross-country athletes may represent a population at high risk of LEA [2]. In fact, a recent review [2] clearly showed that LEA is present among athletes of middle- to long-distance running disciplines, with prevalence ranging from 18% to 58% [11,12,13]. Another recent study conducted by Beermann [9] in 41 collegiate cross-country athletes identified 43.2% of cross-country athletes with clinical LEA. Moreover, elite athletes are exposed to more extreme physical efforts and physiological demands. Hence, it is imperative to characterize elite cross-country athletes to better understand the prevalence of athletes at risk of LEA, as well as to determine which characteristics might be associated with LEA.

Thus, the aim of this study was twofold: (i) To estimate the prevalence of European elite cross-country athletes competing at the 26th European Cross-Country Championships at risk of LEA through a validated questionnaire (i.e., the LEAF-Q); and (ii) to analyze demographic and physical characteristics that are associated with LEA.

## 2. Materials and Methods

The study procedures were approved by the Ethics Committee of NOVA Medical School Faculdade de Ciências Médicas, Universidade NOVA de Lisboa (NMS|FCM) on November 8th of 2019 (102/2019/CEFCM) and on October 22nd of 2019 by the European Athletics, namely the Medical & Anti-Doping Commission. The study was conducted in accordance with the ethical principles expressed in the Declaration of Helsinki for human studies [14], the Portuguese law, and the Good Clinical Practice Guidelines.

### 2.1. Study Design

This study consisted of a cross-section design conducted during the 26th European Cross-Country Championships that took place in Lisbon on 8 December 2019.

All athletes over 18 years old competing at the 26th European Cross-Country Championships (*n* = 602, 266 female and 336 male athletes) were invited to participate in the study. Athletes competing at the 26th European Cross-Country Championships were considered elite athletes as they were selected by their national athletics federation/association to represent their country to compete at an international competition [15]. Data collection took place during leisure time on the days of the competition period prior to the race day at the hotels where the teams were staying. During these leisure times, the research team approached the athletes and presented the aim and study protocol, as well as further information and explanations that they might have considered relevant. Participants signed an informed consent form and were invited to complete a self-administrated questionnaire. The completion of the questionnaire was performed in that moment, in the present of the researchers.

The questionnaire included a validated questionnaire (i.e., the LEAF-Q), among other questions regarding demographic (sex, age, ethnicity, country of origin, education, training characteristics, internationalizations (i.e., participation in competitions abroad)), and reported anthropometric (height, weight, and if their weight had changed more than 3 kg in the previous 3 months) characteristics relevant to the topic. Regarding the last parameter, a weight change of 3 kg was considered a weight change of more than 5%, as it has been described as substantial weight loss [5]. Given the nature of the data collection, the use of a weight percentage was not practical. Thus, by rounding to units, a value of 3 kg was used to consider a weight change of 5% based on Division I cross-country athlete’s average weight in a previous study [16], which found an average weight of 67.7 kg for male and 53.6 kg for female athletes. Moreover, 3 months were considered, as it has been described that the evaluation of the athlete’s risk assessments should be performed at intervals of 1 to 3 months [5]. Body mass index (BMI) was calculated, and athletes were distributed into the correspondent categories, namely underweight (i.e., <18.5 kg/m^2^) and normal weight (i.e., 18.5 to 24.9). The questionnaire was in English. Thus, only athletes who felt comfortable with the English language were eligible to participate. Moreover, the research team was present to clarify any information or term present in the questionnaire if needed. Additionally, participants could, at any moment, refuse to participate in the study without any kind of loss. The survey was available online, while the informed consent was provided and signed in paper.

### 2.2. The Low Energy Availability in Females Questionnaire (LEAF-Q)

The LEAF-Q has been validated for adult elite female endurance athletes and professional dancers with a sensitivity of 78%, specificity of 90% and a test-retest reliability of 0.79 [7]. It contains a total of 25 questions distributed across 3 separate sections: (1) injuries, (2) gastrointestinal symptoms, and (3) menstrual dysfunction and contraceptive use. A total score of ≥8 classifies female athletes as being at risk of LEA as per the LEAF-Q while a section score of ≥2 for injuries, ≥2 for gastrointestinal function, and ≥4 menstrual dysfunction classifies risk for each section (only for female athletes), as per the LEAF-Q scoring system [7,17].

For male athletes, to date, no specific scoring system has yet been validated. Nevertheless, a research group from New Zealand proposed an adapted cut-off for male athletes [8]. This cut-off is the average score of both injuries and gastrointestinal symptoms categories of female athletes identified at risk of LEA for the same study. This sum was 7.40 (2.23) (ranging from 3.0 to 13.0). Thus, the cut-off score of ≥7 was used to identify male athletes at risk of LEA given that the LEAF-Q does not include decimal numbers. Still, caution should be taken as this proposed model is yet not validated.

### 2.3. Statistical Analysis

The statistical analysis of the data was performed by IBM SPSS Statistics software version 25.0 (IBM SPSS Statistics Corporation, Chicago, IL, USA). The normality of each variable was checked using the Shapiro–Wilk test. Comparisons between groups (i.e., sex and risk of LEA) were performed using independent t-test, Mann–Whitney, or Kruskal–Wallis tests depending on the normality of the variables. The chi-square test was used to verify if there was a significant association between risk of LEA and different groups (i.e., sex and if their weight changed in the previous 3 months). Logistic regression was used to determine the odds of being at risk of LEA for the increase of every unit in the participant’s characteristics such as weight, BMI, age, practice years, weekly practice (hours), and number of internationalizations. Odds ratios and 95% confidence intervals were calculated by reference with the first (lower) category of each variable. Descriptive statistics was performed for the outcome measurements and reported as mean (standard deviation (SD)). For all tests, statistical significance was set as *p* < 0.050.

## 3. Results

### 3.1. Sample Characteristics

From the 602 athletes participating in the 26th European Cross-Country Championships, 223 (37.0%) agreed to participate in this study. From these, 16 were excluded because their surveys were incomplete. Therefore, the final sample comprised 207 participants (83 female and 124 male athletes) from 30 countries, including Portugal (16.6%) Spain (12.1%), Italy (8.2%), Ireland (7.7%), and the United Kingdom (4.8%), among others. Participants were mostly Caucasian (89.4%, *n* = 185), but also included Black (8.7%, *n* = 18), Asian (1.4%, *n* = 3), and Arabic (0.5%, *n* = 1) athletes. The athletes’ descriptive characteristics are presented in Table 1.

Variables regarding sports practice, such as years of practice, weekly hours of practice, and number of internationalizations, did not differ between sex (*p* = 0.508, *p* = 0.996 and *p* = 0.184, respectively). Nearly half the athletes (48.8%, *n* = 101) reported weight change in the previous 3 months. More than half the participants reported attending or having attended university or similar (56.5%, *n* = 117), while the remaining athletes reported completing high school (30.0%, *n* = 62) or middle school (12.1%, *n* = 25). Only three individuals reported never attending school (1.4%), with female athletes reporting higher education level when compared to male counterparts (*p* = 0.014).

### 3.2. Risk of Low Energy Availability

A total of 66 female athletes (79.5%) and 67 male athletes (54.0%) were identified as being at risk of LEA. As for the whole sample, a total of 133 athletes (64.3%) were identified as being at risk of LEA.

Table 2 shows the sum scores obtained in each section, the prevalence of female athletes at risk for each section, the total score for the whole questionnaire, and the prevalence of athletes at risk of LEA.

### 3.3. Association Between Risk of Low Energy Availability and Characteristics

Variables regarding sports practice, such as years of practice, weekly hours of practice, and number of internationalizations, did not differ between athletes at risk of LEA or not at risk (*p* = 0.562, *p* = 0.653 and *p* = 0.997, respectively). On the other hand, athletes at risk of LEA reported significantly less weight (−3.7 (1.3) kg, *p* = 0.006) and lower BMI (−0.52 (0.21) kg/m^2^, *p* = 0.044) when compared to athletes not at risk.

Female athletes were more likely to be at risk of LEA (79.5%) when compared to male athletes (54.0%) (x^2^ (1) = 14.060; *p* < 0.001). Additionally, athletes that reported weight change in the previous 3 months were also more likely to be at risk of LEA (70.8%) when compared to those whose weight did not change (57.4%) (x^2^ (1) = 4.000; *p*=0.045). On the other hand, no significant differences were observed for the risk of LEA regarding BMI categories (*p* = 0.260) and different levels of education (*p* = 0.546).

A significant negative association was found between risk of LEA and age (OR = 0.926, 95% CI: 0.864-0.993; *p* = 0.032), weight (OR = 0.935, 95% CI: 0.901–0.971; *p* = 0.001), and BMI (OR = 0.771 95% CI: 0.622–0.956; *p*=0.018), while no significant association was found for practice years (OR = 1.007, 95% CI: 0.941–1.077; *p*=0.848), hours of weekly practice (OR = 1.004; 95% CI: 0.951–1.060; *p* = 0.878), and number of internationalizations (OR = 1.001: 95% CI: 0.944–1.061; *p* = 0.986). However, when analyzed for each sex, no significant association was found between risk of LEA and weight (for female athletes (OR = 1.032, 95% CI: 0.902–1.182; *p* = 0.645) and for male athletes (OR=0.962, 95% CI: 0.907–1.022; *p* = 0.202)), BMI (for female athletes (OR = 0.972, 95% CI: 0.623–1.515; *p* = 0.899) and for male athletes (OR = 0.845, 95% CI: 0.640–1.117; *p* = 0.237)), and age (for female athletes (OR = 0.916, 95% CI: 0.809–1.037; *p* = 0.165) and for male athletes (OR = 0.934, 95% CI: 0.854–1.021; *p* = 0.135)).

### 3.4. The Low Energy Availability in Females Questionnaire (LEAF-Q): Answers per Section

More than half the athletes reported being absent from training or competition due to injury in the previous year (52.2%, *n* = 108), of which a total of 43 (39.8%) reported being absent for at least 22 days. Additionally, the most reported injuries included tendinopathy (*n* = 23), muscle injury (*n* = 17), stress fractures (*n* = 10), unspecified foot injury (*n* = 9), and knee injury (*n* = 7), among others.

Regarding gastrointestinal function, most athletes reported rarely or never feeling gaseous or bloated (60.4%, *n* = 125), as well as rarely or never getting stomach cramps or aches (74.4%, *n* = 154). Additionally, more than half (54.1%, *n* = 112) reported bowel movements once a week or more rarely, with a higher prevalence among male athletes compared to female counterparts (62.1% and 42.2%, *p* < 0.001).

Almost half the female athletes reported menarche at 15 years or older (43.3%, *n* = 36), while three reported never having menstruated. Nearly half the athletes (48.8%, *n* = 39) reported having normal menstruation, while 33 (41.3%) did not report normal menstruation and 8 (10.0%) reported that they did not know. When asked if their menstruation had ever stopped for at least 3 consecutive months, 32.5% (*n* = 26) reported experiencing it in the past and 25.0% (*n* = 20) were experiencing it at that moment. Additionally, when asked if they had ever experienced changes in their menstruation when their exercise load increased, half (50.0%, *n* = 40) answered positively, with most (65.0%, *n* = 26) reporting cessation of menstruation. Nearly one-quarter of the female athletes reported taking oral contraceptives (20.5%, *n* = 17), of which nearly half (41.2%, *n* = 7) reported using oral contraceptives to regulate their menstrual cycle in relation to performance. Others used them as contraception (29.4%, *n* = 5), to prevent the cessation of menstruation (17.6%, *n* = 3), or to reduce menstrual pain (11.8%, *n* = 2).

**Figure 1:**
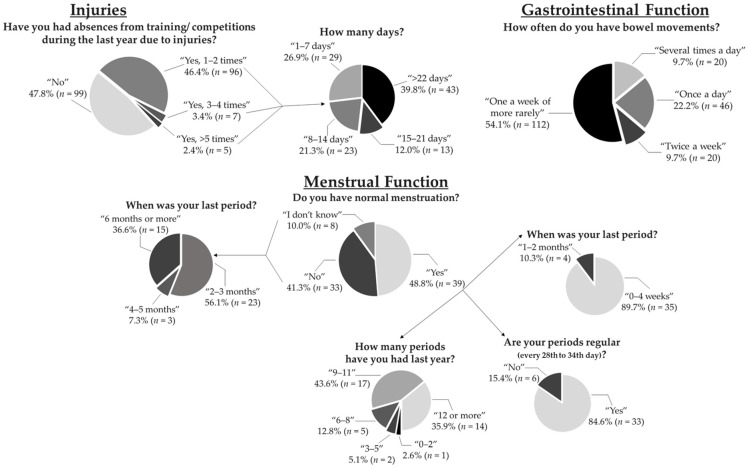
Graphical representation of some answers in the injuries (male and female athletes), gastrointestinal (male and female athletes), and the menstrual function section (female athletes) of the questionnaire.

Further questions and respective answers are presented in Figure 1.

## 4. Discussion

The aim of this study was to estimate the prevalence of European cross-country athletes at risk of LEA using the LEAF-Q [7] and to examine demographic and physical characteristics that are associated with risk of LEA. In our study, we found that the majority of surveyed athletes (64.3%, *n* = 133) were identified as being at risk of LEA. Moreover, risk of LEA was significantly associated with sex (*p* < 0.001), with 79.5% of females (*n* = 66) and 54.0% of males (*n* = 67) identified as being at risk. Age, weight, and BMI were not protective against being at risk of LEA when analyzing separately by sex. The participants of this study were approximately one-third of all the athletes competing at the 26th European Cross-Country Championships. This allowed the collection of data of some of the best elite cross-country athletes in Europe, including various countries, ethnicities, and ages. This provided a major strength to our study as literature is still scarce regarding the application of the LEAF-Q in elite athletes.

### 4.1. Risk of Low Energy Availability

On the one hand, when compared to other studies that assessed the risk of LEA using the LEAF-Q, our results are higher than the ones reported in the majority of previous studies (29.6–63.2%) [17,18,19,20,21,22,23]. However, some studies were conducted in recreational athletes [19,22,23], and none have been conducted in elite cross-country athletes. In a population of female athletes competing in an ultra-marathon (*n* = 306), Folscher et al. [20] observed a prevalence of LEA of 44.1%. However, participants were recruited while they were in line to sign in for the event. Thus, no specification was given about the athletic status (i.e., recreational or elite athletes). Additionally, our findings were closer to the prevalence of athletes at risk of LEA (63.2%) found by Black et al. [23]. However, their study was conducted in recreationally active females (*n* = 38). Hence, given that our sample was composed by elite athletes, this could help explain the higher prevalence we found.

On the other hand, our results seem to extend the findings observed in studies that assessed EA through the aforementioned equation [1]. In a recent study [9] conducted in collegiate cross-country athletes (20 females and 21 males), 82.4% (*n* = 14) females and 80.0% (*n* = 16) males were identified with EA below 45 kcal/kg FFM, of which 41.2% (*n* = 7) females and 45.0% (*n* = 9) males had clinically LEA. Interestingly, in our study, there was a sex difference regarding the risk of LEA, whereas in the study conducted by Beermann et al. [9], no differences were found. However, the authors calculated EA while we estimated the risk of LEA based on reported negatives outcomes of LEA. Additionally, it has been described that male athletes may require lower levels of EA and for longer periods to develop physiological impairments, such as the impairment of bone mineral density [24]. Hence, we might hypothesize that, despite the similar prevalence of athletes with LEA between sex in the study conducted by Beermann et al. [9], the prevalence of symptoms of physiological impairments caused by LEA may differ, leading to a higher prevalence in females than males.

### 4.2. Association Between Risk of Low Energy Availability and Sample Characteristics

When analyzing the associations found for the whole sample, our findings suggest a significant association between being at risk of LEA and age, weight, and BMI. However, these associations are no longer significant when analyzing female and male athletes separately. This may happen because male athletes reported higher values of weight and BMI and had a lower prevalence of risk of LEA. Regarding age, when analyzing female and male athletes, the significant association also disappeared. Hence, when analyzing each sex, age does not appear to affect the risk of LEA.

Our study showed that female athletes had a significantly lower reported weight and BMI than male counterparts, as it has been described in the literature [25,26]. In fact, 40.8% of females reported a BMI lower than 18.5 kg/m^2^, while only 15.4% of males reported a BMI below this threshold. These results are low compared to a group of cross-country athletes from the National Collegiate Athletic Association Division I (21.0 (1.3) kg/m^2^ for males and 20.2 (1.6) kg/m^2^ for females), although BMI in this study did not differ between sex [9]. As expected, sport practice variables did not differ between sex. However, anthropometric differences were found.

### 4.3. Consequences of Low Energy Availability

Increased risk of injuries is one of the sports performance negative outcomes of LEA [4]. In our study, we found that more than half of the athletes reported being absent from training or competition due to injury in the last year. From our findings, we also found that stress fractures were the third most reported injury, with 10 athletes reporting it (6 females and 4 males). Additionally, all athletes that reported stress fractures were identified as being at risk of LEA. Bone stress fractures are considered overuse injuries that occur when decreased bone mineral density and increased porosity lead to the accumulation of microcracks that may consequently lead to bone fractures [27]. Moreover, these stress fractures have already been associated with LEA [28]. Thus, our findings are in line with the increased risk of stress fracture in runners reported in the literature [27].

Our results showed that more than half the participants reported bowel movements once a week or more rarely, indicating a longer-than-expected intestinal transit time. Energy restriction has been suggested to reduce bowel movements and to cause unfavorable changes to the gut microbiota [29], as well as diminished intestinal function and morphologic changes to the gastrointestinal tract [30]. In fact, gastrointestinal symptoms have been linked to athletes with LEA and some other conditions such as disordered eating [31]. Impairments of the gastrointestinal function are also identified as being a health outcome of LEA in the RED-S model, with constipation and increased intestinal transit time identified as main consequences of LEA [4]. Additionally, a study has shown a deleterious effect of LEA on gastrointestinal function compared to those with adequate EA (OR = 1.50, 95% CI: 1.19–1.92; *p* = 0.001) [32]. As mentioned before, male athletes reported a higher prevalence of longer-than-expected intestinal transit time. However, this difference is opposite to what has been observed among nonathletes aged 15 to 50 years, where females tend to show a longer intestinal transit time [33]. On the other hand, constipation has also been linked to dehydration in children and adolescents [34]. Considering that professional athletes frequently present states of hypohydration [35] and that, in weight-sensitive sports, many athletes recur to dehydration to achieve weight loss [36], lower hydration status might also have played a role in explaining our findings.

Disruption of menstrual function was the first identified symptom of a mismatch between EEE and EI [37]. In our study, 39 athletes reported experiencing primary amenorrhea (i.e., menarche at 15 years or more) [32,38]. Although no specific exercise history at an early age was provided, some of the athletes may practice high-intensity exercise from a young age, which could help explain the high prevalence of primary amenorrhea [39], especially if they were involved in weight-sensitive sports such as long-distance running [40]. Moreover, nearly 28% of female athletes (*n* = 23) reported being oligomenorrheic (i.e., cycles at median intervals of at least 35 days [31]), while 22% (*n* = 18) reported experiencing secondary amenorrhea at that moment (i.e., absence of menses for more than 90 days [31]). Regarding menstrual dysfunction, our results seem to be in line with the ranges reported (26% to 43% in female runners) [40]. In our study, 39 female athletes reported experiencing normal menstruation. However, four of those reported experiencing their last menstruation 1 to 2 months prior to the questionnaire, and eight reported less than nine menstruations in the previous year. Hence, this result highlights that caution should be taken when an athlete reports that she is experiencing a ‘normal’ menstruation, as disruption or even absence of menstruation has been wrongfully normalized [40]. It is worth mentioning that 10 athletes reported using oral contraceptive to regulate or even maintain menstruation. As stated in literature [4], combined oral contraceptives should not be used for the resumption of menses. Although exogenous hormones may cause bleeding, unless a spontaneous resumption of menses occurs (i.e., without the use of contraceptives), the deleterious effects of menstrual disruption, such as decreased bone health, continue. Hence, the use of hormonal contraceptives may worsen the global situation by ‘masking’ the continuous decrease of bone mineral density [4]. It should be mentioned that, although no athlete reported the presence of any diseases, we cannot rule out that, without knowing, some of them may have a disease regarding menstrual function that may compromise the regular functioning [31].

### 4.4. Limitations

Although our results provide an important insight about what elite athletes are experiencing regarding EA, as in all studies, some limitations should be addressed. First, we used a proposed scoring system for male athletes that is not yet validated. Although our results seem to corroborate those in the literature, more research is needed to properly validate this option until a male specific questionnaire is available. Hence, caution should be taken when analyzing the findings of the current study, especially regarding the risk of LEA among male athletes. Another limitation is the self-reported nature of the questionnaire as measured values would be preferable. Moreover, the fact that it was applied at a single timepoint is a limitation, as different results may have been seen if administered at different moments of training season. A more detailed body composition analysis (e.g., fat mass and FFM) and blood markers analysis would be ideal, and it would be interesting to conduct future studies in which these parameters are assessed. Another limitation that should be mentioned is that, although the inclusion of athletes from 30 countries provided a wider and possibly a more representative group of participants, this can also increase the group heterogeneity. Additionally, some athletes were not able to participate due to language constraints as they did not speak or read English, the original language of the questionnaire. Last, we should not rule out a misinterpretation of some questions by non-native English speakers.

## 5. Conclusions

In sum, our results clearly demonstrate that elite cross-country athletes represent a population at a high risk of LEA, with a higher prevalence of risk among female athletes. A high prevalence of athletes was identified to have gastrointestinal impairment, with a longer-than-expected transit time. Moreover, a high prevalence of female athletes reported menstrual disfunction. Although in the past, LEA might have been interpreted as a usual and/or needed procedure to achieve better sports performance, nowadays it is recognized that it may lead to several negative consequences. Consequently, more research is needed to further characterize this population and to better understand the risk factors and potential consequences (e.g., gastrointestinal and menstrual impairments). More research will help to easily identify athletes at risk and to clearly and correctly define priorities to achieve a long lasting and successful sports’ career while safeguarding the athlete’s health. Nevertheless, the data from the present study will allow the development of educational programs specifically directed to European athletes. Moreover, these athletes should be followed by a multidisciplinary team in an effort to prevent, educate, treat, or minimize LEA and its effects on the body physiological functioning.

## Figures and Tables

**Table 1:** Anthropometric and training characteristics of the sample and divided by sex.

	Whole Sample (*n* = 207)			Female (*n* = 83)			Male (*n* = 124)			*p*
	Mean	SD	Range	Mean	SD	Range	Mean	SD	Range	
Age (years)	22.1	4.0	18.0–35.0	21.8	4.0	18.0–35.0	22.3	4.1	18.0–35.0	0.320
Weight (kg)	58.4	8.0	43.0–76.0	51.0	4.1	43.0–61.0	63.2	6.1	46.0–76.0	<0.001
Height (cm)	173.9	8.9	154.0–196.0	165.8	5.1	154.0–176.0	179.2	6.6	163.0–196.0	<0.001
BMI (kg/m^2^)	19.3	1.4	15.7–24.2	18.6	1.3	16.2–22.4	19.8	1.3	15.7–24.2	<0.001
Years of practice	7.2	4.3	0.0–20.0	7.0	4.1	0.0–20.0	7.4	4.3	0.0–19.0	0.508
Weekly practice (h)	14.8	5.4	5.0–40.0	14.8	5.4	6.0–40.0	14.7	5.5	5.0–35.0	0.996
Internationalizations	4.5	4.9	0.0–40.0	6.4	4.5	0.0–34.0	4.4	5.1	0.0–40.0	0.184

SD: Standard deviation; BM: Body Mass Index; Years of practice: Number of years the athlete was enrolled in cross-country; Internationalizations: Number of participations in competitions abroad; *p*: Level of significance of the difference between sex with the t-test for variables with normal distribution (i.e., height and weight) or the Mann–Whitney test for variables without normal distribution (i.e., age, BMI, years of practice, weekly practice, internationalizations). Significance set at *p* < 0.050.

**Table 2:** Sum score and prevalence of athletes at risk for each section and total Low Energy Availability in Females Questionnaire (LEAF-Q).

	Whole Sample (*n* = 207)		Female(*n* = 83)		Male (*n* = 124)		*p*
	Mean (SD)	*n* (%)	Mean (SD)	*n* (%)	Mean (SD)	*n* (%)	
Injury section	3.0 (2.2)	N/a	3.1 (2.2)	46 (55.6)	2.9 (2.2)	N/a	0.395
Gastrointestinal section	3.9 (1.9)	N/a	3.5 (2.1)	62 (74.7)	4.1 (1.7)	N/a	0.038
Menstrual function and contraceptive use section	N/a	N/a	5.4 (3.2)	59 (71.1)	N/a	N/a	N/a
Total	N/a	133 (64.3)	12.0 (4.4)	66 (79.5)	7.0 (2.9)	67 (54.0)	<0.001

SD: Standard deviation.; *n* (%): Number and percentage of athletes identified as being at risk: injury risk score ≥ 2 (only for female athletes), Gastrointestinal risk score ≥ 2 (only for female athletes), Menstrual function and contraceptive use section risk score ≥ 4 (only for female athletes), Total score (i.e., risk of low energy availability) risk score ≥ 8 or ≥ 7 for female or male athletes, respectively; *p*: Level of significance of the difference between sex with the independent t-test for variables with normal distribution (i.e., total score) or the Mann–Whitney test for variables without normal distribution (i.e., injury section score and gastrointestinal section score); N/a: Not applicable. Range scores for whole sample were 1.0 to 8.0 for the injury section and 0.0 to 9.0 for the gastrointestinal section. Range scores for female athletes were 1.0 to 8.0 for the injury section, 0.0 to 9.0 for the gastrointestinal section, 0.0 to 11.0 for the contraceptive and menstrual function section, and 3.0 to 21.0 for the total score. Range scores for male athletes were 1.0 to 8.0 for the injury section, 0.0 to 7.0 for the gastrointestinal section, and 1.0 to 14.0 for the total score. Significance set at *p* < 0.050.

## Data Availability

The data for the current study is available within this manuscript.
